# Indirect, machine learning-based suicide risk screening: Evidence from cross-National Validation

**DOI:** 10.1192/j.eurpsy.2026.10166

**Published:** 2026-02-13

**Authors:** Polona Rus Prelog, Martina Rojnic Kuzman, Teodora Matić, Peter Pregelj, Sara Medved, Sarah Bjedov, Irena Rojnic Palavra, Anamarija Petek Eric, Stipe Drmic, Domagoj Vidovic, Aleksander Sadikov

**Affiliations:** 1 University Psychiatric Clinic of Ljubljana: Univerzitetna psihiatricna klinika L, Slovenia; 2 University of Ljubljana Faculty of Medicine: Univerza v Ljubljani Medicinska Fak, Slovenia; 3 University of Zagreb School of Medicine: Sveuciliste u Zagrebu Medicinski fakult, Croatia; 4 University of Ljubljana Faculty of Computer and Information Science: Univerza v, Slovenia; 5 University Hospital Centre Zagreb: Klinicki Bolnicki Centar Zagreb, Croatia; 6 Jankomir Psychiatric Hospital: Psihijatrijska Bolnica Sveti Ivan, Croatia; 7 University Hospital Centre Osijek: Klinicki bolnicki centar Osijek, Croatia; 8 Dubrava Clinical Hospital: Klinicka Bolnica Dubrava, Croatia; 9 University Psychiatric Hospital Vrapče: Klinika za psihijatriju Vrapce, Croatia

**Keywords:** machine learning, mass screening, risk assessment, suicidal ideation

## Abstract

**Background:**

Suicide is a major public health challenge requiring early detection of suicidal ideation (SI). Traditional direct questioning methods suffer from stigma and disclosure bias, failing to identify many at-risk individuals. While machine learning (ML) models show promise, most lack external validation. Indirect screening, using psychosocial data rather than direct SI questions, offers a scalable alternative. This study aimed to externally validate an indirect, ML-based SI screening tool. We tested if a model trained on a Slovenian general population sample retained predictive accuracy when applied to an independent Croatian sample during a period of societal stress (pandemic and earthquakes), assessing performance across age and gender subgroups.

**Methods:**

A logistic regression model was trained on a Slovenian sample (*N* = 2,989) and validated on a Croatian sample (*N* = 2,364). The model used only indirect predictors, including sociodemographics, life satisfaction, behavioral changes, and Brief COPE subscales. The target outcome was the presence of SI (SIDAS score > 0). Performance was measured by the area under the receiver operating characteristic curve (AUROC).

**Results:**

The model demonstrated strong external validity on the entire Croatian sample, achieving an AUROC of 0.80. Performance remained robust across subgroups: males (AUROC = 0.83), females (AUROC = 0.79), younger adults (AUROC = 0.77), and older adults (AUROC = 0.81). Self-blame, behavioral disengagement, and relationship dissatisfaction were key predictors.

**Conclusions:**

An indirect, ML-based screening tool can reliably identify SI risk in the general population. The model demonstrated strong cross-national transferability and resilience during a societal crisis, proving it is a feasible and valid strategy for population-level prevention.

## Background

Suicide remains a major public health problem throughout Europe, accounting for tens of thousands of deaths annually [1] and reflecting deep inequities in mental health outcomes across populations. The early detection of suicidal ideation (SI), a critical precursor to suicide attempts and fatalities, is widely recognized as the cornerstone of suicide prevention efforts. However, scalable and equitable methods for SI risk identification are not yet implemented in routine population health surveillance, in part due to stigma, limited acceptability of direct questioning, and variable access to mental health care [2].

Recent advances in machine learning (ML) and artificial intelligence (AI) have revolutionized risk prediction, demonstrating that models can leverage large-scale, multidimensional data—including psychosocial, behavioral, and social factors—to achieve strong and sometimes superior predictive performance compared to traditional clinical algorithms. In a meta-analysis of adolescent suicide risk, for instance, ML-based models achieved pooled AUCs between 0.77 and 0.84, with substantial improvement over traditional models and promising utility for digital population health [2]. Similar findings were observed in late-life suicide, where connectedness and social belonging, captured via proxy variables, emerged as dominant ML predictors [3]. In line, a recent comprehensive review highlighted the expansion of ML to psychiatric and population settings, while stressing the urgent need for external validation and focus on real-world implementation across age groups and regions [4].

Despite these advances, the transferability and robustness of SI prediction models across populations, languages, and under diverse conditions, like acute societal stressors—such as disasters, pandemics, war etc. – remain under-studied. Most published models are derived and validated in single-country data or clinical samples [5, 6], with only a handful exploring external national validation or cross-context resilience [7]. Moreover, high-impact research shows that the predictors of suicide and SI – such as social isolation, chronic illness, and emotion regulation – may vary across demographic subgroups, making age- and gender-sensitive model reporting essential for both statistical validity and health equity [3, 6]. Integration of these principles is increasingly prioritized in global and European mental health policy [5, 7].

Moreover, traditional suicide risk screening in both clinical and community settings has largely relied on direct questioning about self-harm thoughts and intentions, using instruments such as the Columbia Suicide Severity Rating Scale (C-SSRS) [8] or the Suicidal Ideation Attributes Scale (SIDAS) [9]. However, direct questioning may lead to disclosure bias – even in anonymous surveys – and raises serious ethical and acceptability issues, especially outside specialized psychiatric care settings. Many individuals at risk, particularly in the general population, may be unwilling to answer direct suicidality questions truthfully or may be deterred from participating in screening altogether. Moreover, most current approaches – and nearly all high-performing clinical prediction tools – focus narrowly on populations already identified as having mental health struggles, such as those with known depression, prior attempts, or who are actively seeking psychiatric care. This focus risks missing a significant segment of the population whose suicidal ideation may arise in the absence of diagnosed mental disorders, particularly in periods of heightened psychosocial stress.

Our recent work demonstrates that screening for SI *without* reference to suicidal thoughts, mood, or prior psychiatric history – by using only indirect questions about daily habits, satisfaction, and coping strategies, can reliably identify at-risk individuals in the general population [10]. Such approaches have been shown to perform on par with or better than traditional direct-question methods, while reducing participant burden and minimizing non-response or misreporting related to the stigma of suicidality screening [11]. Indirect, ML-based tools thus offer a realistic path to broader SI detection – enabling integration into general health check-ups, occupational medicine, and possibly even digital self-assessment platforms. Crucially, these tools have the potential to detect those *without* existing mental health diagnoses or clinical histories and for implementation for public health surveillance and selective interventions.

### Objective

Therefore, in this study, our aim was to evaluate whether an ML-based, *indirect* screening tool for SI – originally developed in a large sample of Slovenian general adult population – retains external validity when applied to Croatian adult population, including during a period of elevated societal stress (pandemic, earthquake) and to explicitly analyze the model’s performance not only in the total population but also within key age and gender subgroups, to enable the development of future suicide prevention strategies for the general population.

## Methods

### Study design and sample characteristics

The data used for training and validating machine learning models was obtained from an ongoing population-based study, which forms part of a larger international multicenter research initiative [12]. Data collection was conducted via an online questionnaire administered to a nationwide community sample of adults in Slovenia and Croatia. A more detailed description of the implementation procedure is available in both cited studies [10, 11]. The study received ethical approval from the Republic of Slovenia National Medical Ethics Committee (protocol no. 0120–283/2020/7) and Croatian Psychiatric Association (protocol no. 1/2020) and was carried out in accordance with relevant guidelines and regulations, including the Declaration of Helsinki. Informed consent for participation was obtained from all participants using the online form prior to the beginning of the study questionnaire. Data collection in Slovenia spanned from July 23, 2020, through February 23, 2022, while in Croatia, the data was collected from April 26, 2020, through August 7, 2020. The total Slovenian sample included 2,989 participants – 1,790 in the first COVID-19 wave and 1,199 in the second wave, while the Croatian sample included a total of 2,364 participants. The full sample from Slovenia was used for model training, resulting in the training set of 2,989 participants. The model was tested on the full Croatian sample of (2,364 participants), as well as on subgroups: males (668 participants), females (1,696 participants), younger people (defined as ≤32 years old; 700 participants) and older people (defined as >32 years old; 1,664 participants).

### Measures

A broad set of predictors was employed in the machine learning setup, which have been described in detail in our original paper [10].

Sociodemographic variables included age, gender, and the number of household members. Life satisfaction was assessed using three questions targeting satisfaction with: (1) financial situation, (2) relationships with household members or closest people if living alone, and (3) housing. Each was rated on a 7-point Likert scale ranging from “could not be worse” to “could not be better.” These items were treated as individual variables, as they do not constitute a predefined scale.

Behavioral change variables included increased use of substances (tobacco, alcohol, marijuana, other psychoactive substances, and non-prescribed drugs), changes in physical activity and eating (both increase or decrease), and the estimated daily amount of time spent online.

Stress coping was measured using the Brief COPE inventory [13], a 28-item multidimensional tool assessing the frequency of use of various coping strategies on a 4-point scale ranging from 1 (“I haven’t been doing this at all”) to 4 (“I’ve been doing this a lot”). We included all 14 subscales as individual variables: (1) self-distraction, (2) active coping, (3) denial, (4) substance use, (5) use of emotional support, (6) use of instrumental support, (7) behavioral disengagement, (8) venting, (9) positive reframing, (10) planning, (11) humor, (12) acceptance, (13) religion, and (14) self-blame. Each subscale score was computed by summing its two respective items.

Suicidal ideation (SI) was measured using the Suicidal Ideation Attributes Scale (SIDAS) [9]. This instrument comprises five items assessing frequency of suicidal thoughts, controllability, closeness to attempt, associated distress, and interference with daily functioning over the past month. Each item is rated on a 10-point Likert scale, producing a total score between 0 and 50. If a participant selected “0—Never” on the first item, the remaining items were skipped, and the total score was recorded as zero. Given that any indication of SI suggests a potential suicide risk [9], we categorized the sample into those scoring zero (no SI) and those scoring above zero (presence of SI in the past month).

### The models

Our original model for predicting suicidal ideation (SI) and its validation can be found in [10, 11]. The original model was trained on the 70% of the data from the first wave of the COVID-19 pandemic (1,253 respondents), stratified by SI (the target variable), and depression level, while the remaining 30% (537 respondents) were set aside for testing. Further independent validation was done using the later (post-pandemic, 2022) data (1,199 respondents). Four machine learning algorithms were employed (logistic regression (LGR), random forest, XGBoost, and support vector machines), but we reported the results of the LGR, because it slightly outperformed the other models and especially for its ease of interpretation.

In this paper, we re-trained the model again using LGR and the same set of predictors, this time on the entire set of data collected in Slovenia. To test the model’s transferability, we tested the model on the data collected in Croatia, both the complete sample, and age groups (≤32 years old and > 32 years old) and genders (male and female) separately.

The models used the following predictors: (1) a set of sociodemographic variables (age, gender, and the number of people living in the household), (2) satisfaction of with three aspects of life – personal financial situation, relationship with the people the person lives with, or relationships with their closest people if they lived alone, and their current housing (these three questions used as individual variables as they do not form a predefined scale), (3) a set of variables related to recent changes in behavior (increase in substance use – any among tobacco, alcohol, marijuana, other psychoactive substances, and drugs not prescribed by a physician, quantitative change in the level of physical activity and eating, and the estimated daily amount of time spent online), and (4) the Brief COPE scale with all of its 14 subscales used as separate predictors.

The target variable was the binarized SIDAS (zero or above zero).

## Results

The sample characteristics for the complete Slovenian and Croatian samples are given in Table 1, while the descriptive statistics of the subgroups of the Croatian sample used for testing are provided in Table 2.

**Table 1.** Sample characteristics

Metric		Slovenian sample	Croatian sample
Age	Mean (± SD)	38.7 (± 12.6)	40.2 (± 12.2)
	Median	38	40
Gender	% Female	79.09	71.74
Education	Incomplete primary (%)	0.03	-
	Primary (%)	-	0.51
	Secondary (%)	24.24	24.28
	University (%)	75.73	75.21
Marital status	Married/cohabiting (%)	65.47	61.17
	Divorced (%)	2.81	7.28
	Single (%)	19.20	18.82
	Widowed (%)	0.70	1.23
	In relationship (%)	11.81	11.51
SIDAS	% Above 0	13.25	12.27

**Table 2.** Subgroup characteristics of Croatian sample

Metric		Gender		Age group	
		Male	Female	Young	Old
Age	Mean (± SD)	40.2 (± 12.2)	40.3 (± 12.2)	26.0 (± 3.9)	46.2 (± 9.3)
	Median	39	40	26	44
Gender	% Female	-	100.00	71.00	72.06
	Primary (%)	0.15	0.65	0.86	0.36
	Secondary (%)	26.80	23.29	36.57	19.11
	University (%)	72.65	76.06	62.58	80.53
Marital status	Married/cohabiting (%)	60.48	61.44	29.71	74.40
	Divorced (%)	4.49	8.37	1.57	9.68
	Single (%)	22.75	17.28	39.57	10.10
	Widowed (%)	0.30	1.59	-	1.74
	In relationship (%)	11.98	11.32	29.14	4.09
SIDAS	% above 0	14.22	11.50	20.43	8.83

The complete set of regression coefficients for the updated model, and the original model described in (10) is provided in Table 3.

**Table 3.** Regression coefficients (after intercept) sorted in descending order by the absolute value (importance) in the updated model

Feature	Updated model exp. coefficient	Original model exp. coefficient
Intercept	0.20	0.18
BC self-blame	9.00	7.75
BC behavioral disengagement	3.56	3.43
BC substance use	3.17	4.37
BC self-distraction	2.27	1.68
Daily number of hours spent online	2.26	1.08
BC denial	2.10	1.78
Age	0.24	0.50
BC religion	1.68	1.48
BC positive reframing	0.32	0.26
Satisfaction with relationships	0.33	0.43
BC humor	1.60	1.20
BC emotional support	0.52	0.97
BC active coping	0.52	0.73
BC venting	1.41	1.48
BC instrumental support	0.62	0.84
Substance use (no increase)	0.69	0.66
Gender (male)	1.26	1.41
Number of people in the household	0.76	0.91
BC acceptance	0.78	0.86
Satisfaction with finance	0.80	0.88
Change in physical activity (no change)	0.86	0.78
Satisfaction with housing	1.08	0.55
BC planning	1.00	0.85
Change in eating habits (no change)	1.00	1.10

**Figure 1.**
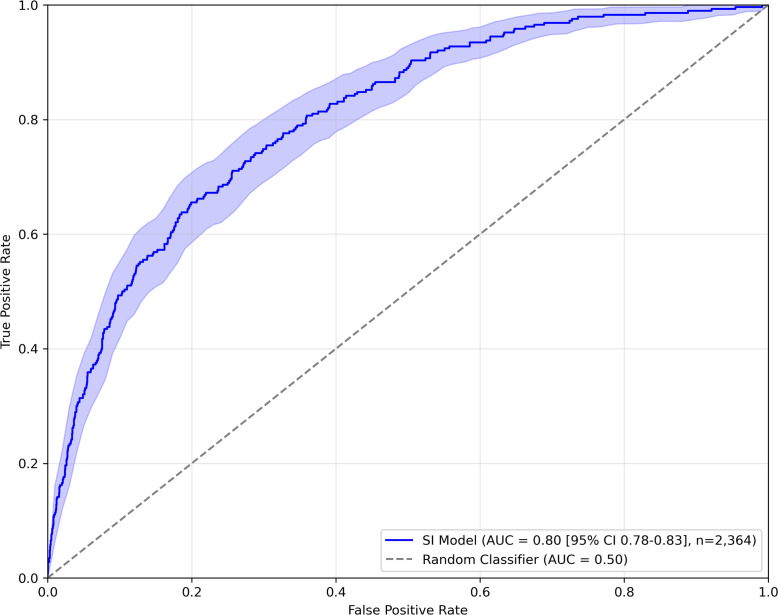
ROC curves of model predicting SI on complete Croatian sample.

We tested the model on the complete Croatian data set (2,364 participants) and observed area under the Receiver Operating Characteristic curve (AUROC) of 0.80. Receiver Operating Characteristic (ROC) curve is presented in Figure 1.

**Figure 2.**
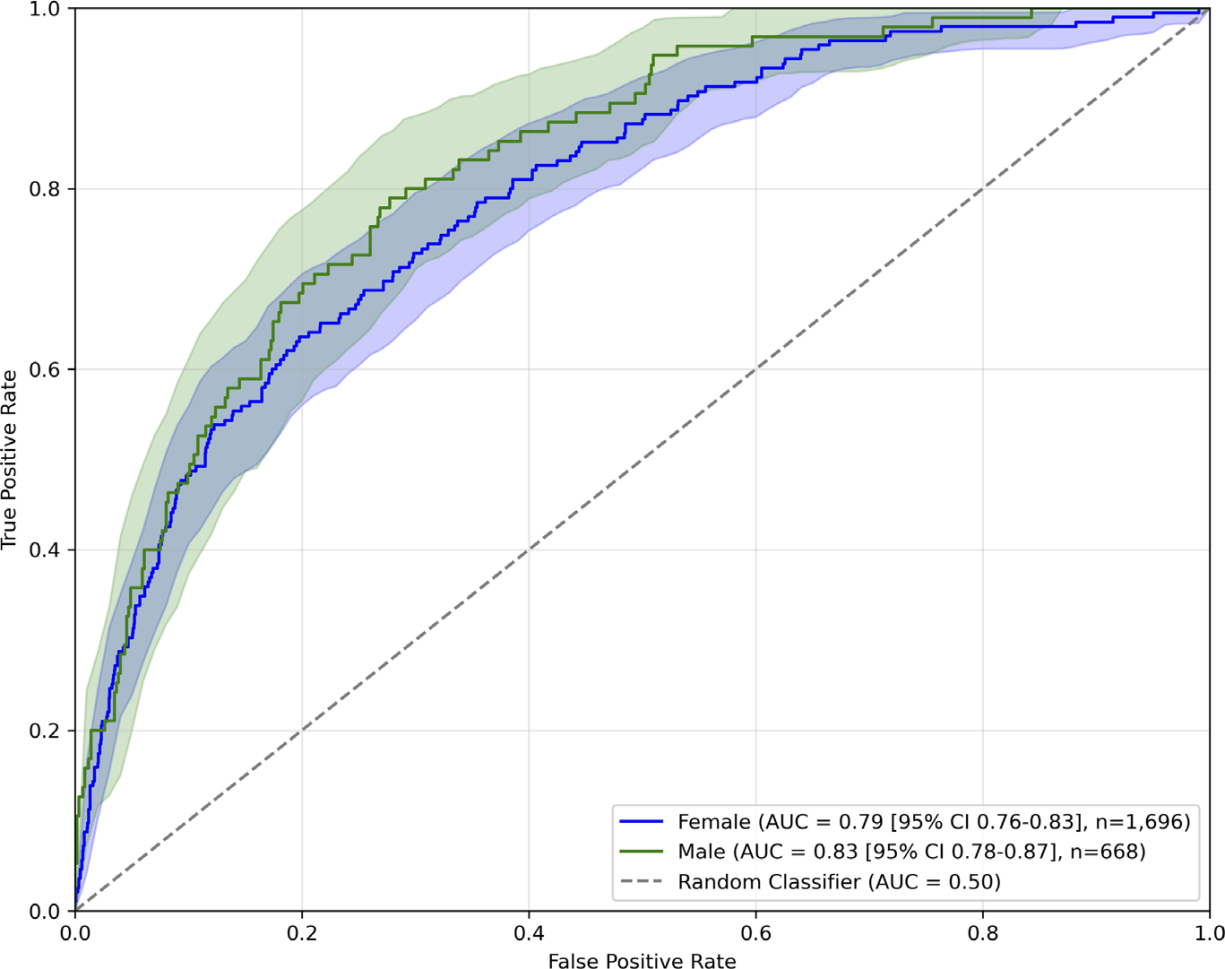
ROC curves of model predicting SI on Croatian sample, for each gender separately.

On the subset of males, we observed the AUC of 0.83, while in the subset of females it was 0.79. ROC curves are presented in Figure 2.

**Figure 3.**
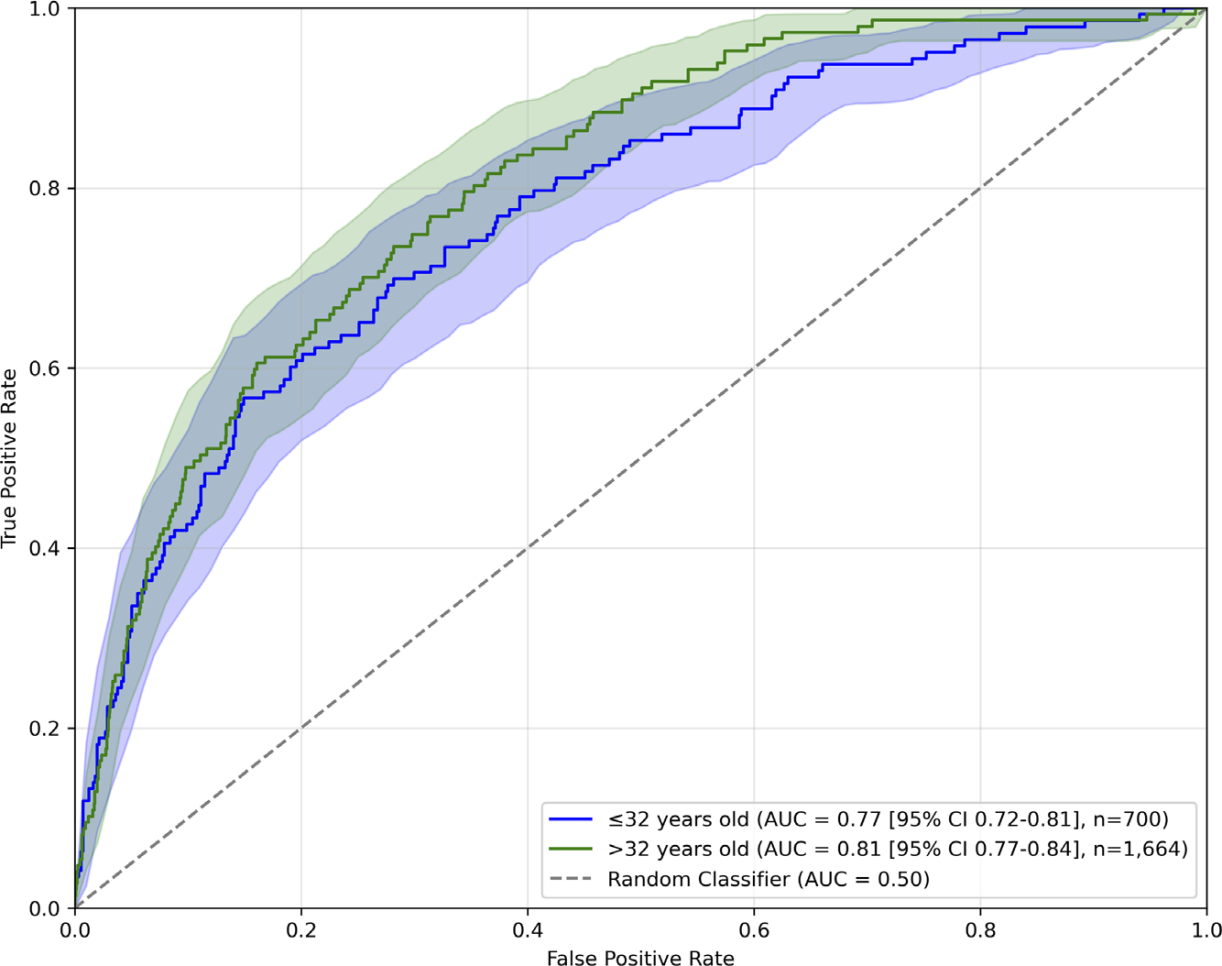
ROC curves of model predicting SI on Croatian sample, for each age group separately.

On the subset of younger participants, we observed the AUC of 0.77, while in the subset of older participants it was 0.81. ROC curves are presented in Figure 3.

## Conclusions

This study provides strong, externally validated evidence that indirect, machine learning–based screening with our model – using only psychosocial, behavioral, and demographic data – can identify individuals at risk for SI in the general population, without the need for explicit questions about suicide or a focus on known mental health cases. Unlike most previous work, which has been limited to direct risk instruments or clinical samples, we show that such a model, developed on Slovenian population, retains robust performance (AUC = 0.80) when deployed in a fully independent cohort from Croatia, including under the slightly different real-world challenge of natural disaster stress. These results underscore the potential of indirect, low-burden digital screening to transform suicide prevention as a tool for all, not just for psychiatric populations.

Our previous work developed a machine learning model using only indirect questions (coping, satisfaction, habits) in the Slovenian general population, reporting strong AUROC performance. When tested for validation during a period with restored social circumstances (the latter, post-pandemic timeframe), model performance showed minimal decline even in the presence of data drift, reinforcing its robustness in “normal” as well as crisis conditions [10, 11]. Importantly, the principal aim of the present study was to externally validate such an indirect approach by applying a model (trained on a Slovenian population sample) to a fully independent Croatian national sample, thus directly testing cross-country generalizability. Our results confirm strong discriminatory power not only for the population as a whole, but also within age and gender subgroups, underlining the robustness and transferability of this methodology.

Our model’s discriminatory power (AUC = 0.80 on Croatian sample; AUC > 0.76 for all gender/age subgroups) parallels or exceeds other recent European ML models for suicide risk, which typically report AUCs between 0.71 and 0.82 [2, 4]. Behavioral disengagement, self-blame, substance use, coping, and social dissatisfaction repeatedly emerged as the strongest risk factors, mostly aligning with established suicide theories and population studies across Europe [3, 14]. The observed protective effects of positive reframing and older age are congruent with resilience literature and recent cross-national meta-analyses [15, 16]. Crucially, this model maintained its accuracy after “real-world” transfer to a Croatian population exposed during the study period to a “double disaster” – pandemic and major earthquakes (22nd of March 2020 and 29th of December 2020), a factor known to elevate SI risk and pressure health systems [17, 18], with several studies showing elevated general mental distress, aggravation of the use of substances and general health in non-clinical [19] and clinical population in Croatia [20, 21].

However, only a few of the existing risk models have tested validity under such acute, extrinsic stressors, considerably extending the generalizability, and resilience evidence base [22]. Prior ML studies in Europe, including Swedish and UK Biobank analyses, predominantly focused on registry or electronic health record data and often require direct disclosure of SI or diagnosis [23, 24]. Our approach, employing only indirect, non-clinical predictors, demonstrates comparable or improved risk stratification with reduced potential for stigma and broader community applicability – particularly for those less likely to engage with clinical services, such as men and younger adults.

Notably, subgroup performance proved quite robust, echoing findings from pan-European suicide research that highlight the need for gender- and age-sensitive models [3, 25]. Our study adds to the limited number of true “external validations” in this field – a critical methodological standard often lacking but increasingly mandated by leading journals and policy consortia [26].

Our findings also align with recent policy calls for integrated digital solutions to European mental health challenges, especially post-pandemic and during protracted crises. Scalable, ML-driven screening can support resource prioritization and facilitate early intervention in under-resourced regions—a central theme across recent The Lancet Regional Health – Europe publications and others [27].

Other ML studies have shown that population-based, indirect approaches can match or exceed the prediction accuracy of direct clinical tools. For example, a Korean general population study integrating sociodemographic and behavioral data achieved an AUC of 0.85 [28], while a US NHANES-sample ML study obtained similar results using public health survey data [29]. Reviews and systematic analyses likewise affirm that most ML models using survey or administrative data yield AUCs in the 0.75–0.86 range [2]. A critical advance of our research is the demonstration that our models retain performance in external, cross-country samples under acute environmental stress (pandemic and earthquake in Croatia). Most prior tools, and virtually all clinical algorithms, have not been systematically tested under such real-world, non-clinical, population-health circumstances [29].

Moreover, traditional SI screening methods, such as the C-SSRS or patient health questionnaires, require both willingness to disclose suicidality and the ability to recognize one’s own risk – barriers that lead to high rates of non-disclosure and missed cases in community and even clinical settings [11]. By using only indirect questions – like stress coping, changes in daily habits, and social satisfaction – our approach aligns with emerging evidence that a substantial portion of SI occurs outside the realm of diagnosed mental illness or active treatment [29] and that indirect approaches can yield comparable, if not better, prediction accuracy, offering a path toward more scalable, stigma-resistant suicide prevention.

Indirect methodology also greatly expands the reach of suicide risk assessment. A recent systematic review underscores that indirect models, trained with population-level data, can be rapidly deployed online, minimize stigma, and achieve high participation rates in diverse groups [7]. The model, which omits any direct SI queries, maintained high sensitivity and specificity even across different languages, social contexts, and in subgroups with traditionally lower health-seeking (men, young adults), confirming findings by other researchers [3, 28].

A unique feature of our study is the rigorous, cross-country external validation. While many studies have reported promising ML results in development samples, very few have taken the crucial step of testing their models in wholly independent, contextually distinct populations [4]. Here, the model trained on Slovenian data performed nearly identically when applied to Croatia. The model was trained on the entire set of data collected in Slovenia (which included the pandemic and post-pandemic time frame, when circumstances returned to normal) and tested on Croatian data, collected during the pandemic, but also during the extraordinary context of widespread earthquake-related trauma [21, 30]. This finding suggests that the model works in different circumstances, but also that psychosocial risk pathways for SI are sufficiently universal to allow robust, transferable screening – an insight with broad relevance for European and global health systems, particularly in an era of different crisis-driven stressors.

Importantly, our results also confirmed robust model performance across age and gender groups, helping address longstanding gaps in population suicide prevention. Our indirect tool has the potential to offer a scalable solution for occupational or community programs, and for further integration into digital health records, primary care triage, and public platform self-assessment tools. This path aligns closely with current recommendations for digital, upstream prevention from WHO Europe, the EU, and leading suicide prevention experts [2, 3].

### Strengths and limitations

A major strength of our work is the use of true, cross-national external validation in a large, population-based national sample during an episode of societal emergency. Another strength is the comprehensive inclusion of modifiable, non-clinical predictors. This supports the model’s generalizability – a step seldom addressed in the literature. Comprehensive evaluation by age and gender further demonstrates its potential for valid screening.

However, several limitations should be noted; cross-sectional and self-reported data may always introduce recall and response bias. The absence of longitudinal validation limits inferences about the prediction of future SI or suicide attempts. Next, digital recruitment and online data collection carry the risk of underrepresented high-risk groups with poor digital access, such as elderly or digitally illiterate or those with severe illness. Furthermore, while indirect predictors cover broad domains, some relevant factors (e.g., trauma, acute life events) may be underrepresented. Finally, to promote generalizability beyond Europe, other populations should be studied, in economically, linguistically, and culturally diverse regions [7], especially considering the possibly similar regional, historical, and culturological factors in the two studied countries.

A part of the Slovenian and a large part of Croatian datasets were collected during the acute phase of the COVID-19 pandemic (the latter sample including the earthquake timeframe), a period marked by unprecedented societal stress, social isolation, and economic uncertainty. This context likely influenced the prevalence of SI in the population and our sample – but at the same time allowed for sufficient SI positives for the model’s training. However, while our results enhance the external validity for pandemic and other crisis situations, they also introduce a potential confounder due to transient pandemic-specific factors rather than persistent features of risk in stable periods.

Our findings, in the context of converging evidence from multiple countries, support indirect, ML-based screening as a feasible and equitable option for suicide prevention in the general population. The next generation of research should focus on further international settings, longitudinal validation, integration with digital health systems, continual updating for changing social conditions, and ensuring accessibility for disadvantaged groups. As the global population’s mental health faces mounting stress, such models offer practical potential to close the gap between clinical knowledge and public health action. Taken together, these findings suggest that indirect ML models offer a promising pathway to broader, more equitable suicide prevention in the general population. By sidestepping the stigma and non-response associated with direct SI questions, these models could be well-suited for digital self-assessment and community surveillance settings and may be especially useful to reach subpopulations (men, young adults) less likely to seek mental health care or disclose suicidality [4]. Importantly, our models rely on universally accessible, non-intrusive survey items, facilitating possible implementation at national scales and in various languages. Their integration into online health portals or primary care workflows could substantially lower the threshold for timely SI risk identification and support targeted intervention where it is most needed.

## Data Availability

Data from the current study can be made available upon request to the corresponding author.
